# The Widely Used ATB FUNGUS 3 Automated Readings in China and Its Misleading High MICs of *Candida* spp. to Azoles: Challenges for Developing Countries' Clinical Microbiology Labs

**DOI:** 10.1371/journal.pone.0114004

**Published:** 2014-12-02

**Authors:** Li Zhang, He Wang, Meng Xiao, Timothy Kudinha, Lei-Li Mao, Hao-Ran Zhao, Fanrong Kong, Ying-Chun Xu

**Affiliations:** 1 Department of Clinical Laboratory, Peking Union Medical College Hospital, Chinese Academy of Medical Sciences, Beijing 100730, P. R. China; 2 Graduate School, Peking Union Medical College, Chinese Academy of Medical Sciences, Beijing 100730, P. R. China; 3 Centre for Infectious Diseases and Microbiology Laboratory Services, Westmead Hospital, Darcy Road, Westmead, New South Wales 2145, Australia; V.P.Chest Institute, India

## Abstract

The rapid development in the clinical microbiology diagnostic assays presents more challenges for developing countries than for the developed world, especially in the area of test validation before the introduction of new tests. Here we report on the misleading high MICs of *Candida* spp. to azoles using the ATB FUNGUS 3 (bioMérieux, La Balme-les Grottes, France) with automated readings in China to highlight the dangers of introducing a diagnostic assay without validation. ATB FUNGUS 3 is the most commonly used commercial antifungal susceptibility testing method in China. An in-depth analysis of data showed higher levels of resistance to azoles when ATB FUNGUS 3 strips were read automatically than when read visually. Based on this finding, the performance of ATB FUNGUS 3, read both visually and automatically, was evaluated by testing 218 isolates of five clinically important *Candida* species, using broth microdilution (BMD) following CLSI M27-A3 as the gold-standard. The overall essential agreement (EA) between ATB visual readings and BMD was 99.1%. In contrast, the ATB automated readings showed higher discrepancies with BMD, with overall EA of 86.2%, and specifically lower EA was observed for fluconazole (80.7%), voriconazole (77.5%), and itraconazole (73.4%), which was most likely due to the trailing effect of azoles. The major errors in azole drug susceptibilities by ATB automated readings is a concern in China that can result in misleading clinical antifungal drug selection and pseudo high rates of antifungal resistance. Therefore, the ATB visual reading is generally recommended. In the meantime, we propose a practical algorithm to be followed for ATB FUNGUS 3 antifungal susceptibility for *Candida* spp. before the improvement in the automated reading system.

## Introduction

The rapid development in clinical microbiology laboratory diagnostic assays presents more challenges for developing countries than their developed world counterparts, especially with regard to validation and quality control of such assays. The widely used but problematic ATB FUNGUS 3 with ATB Expression Bacteriology Analyzer automated readings (bioMérieux, La Balme-les Grottes, France) in China and its misleading reported high MICs of *Candida* spp. to azoles, gave a very good show case to highlight the challenges faced by clinical microbiology labs in developing countries.

Invasive candidiasis is now widely recognized as an important public health problem, with considerable morbidity, mortality, and associated health care costs [1]–[3]. Antifungal surveillance programs like ARTEMIS Global Antifungal Surveillance Program play an important role in the management of fungal infections, guiding clinical treatment as well as tracking the development of drug resistance [4]–[5].

Unlike in the developed world, multicenter and long term antifungal surveillance data is lacking in China. Furthermore, an analysis of reported data on antifungal susceptibility patterns from different centers in China revealed considerable variability, especially for azole drugs. ATB FUNGUS 3 is the most commonly used commercialized antifungal susceptibility test method in China, and an in-depth analysis of data on *Candida* susceptibility to fluconazole showed higher levels of resistance when ATB FUNGUS 3 strips were read automatically than when read visually, suggesting possible errors by the ATB FUNGUS 3 strips automated reading system [6]–[10].

Thus, the main aim of the present study was to evaluate the performance of the ATB FUNGUS 3 strips (both read visually and automatically) in relation to the Clinical and Laboratory Standard Institute (CLSI) broth microdilution (BMD) method for the *in vitro* antifungal susceptibility testing of *Candida* isolates from multicenters in China. A secondary aim was to develop a practical and efficient algorithm for *Candida* species susceptibility testing by ATB FUNGUS 3 strips, using a combination of visual and automated readings.

## Materials and Methods

### 1. *Candida* isolates

A total of 218 isolates of five clinically important *Candida* species selected from the 2010 CHIF-NET program from 12 study centers (August 2009–July 2010) were used [11]. All the resistant isolates of the five *Candida* species were included in the study, whilst the susceptible isolates were selected randomly. These isolates included 81 isolates of *Candida albicans*, 42 of *Candida glabrata*, 41 of *Candida tropicalis*, 38 of *Candida parapsilosis* complex (20 of *C. parapsilosis* sensu stricto, 14 of *Candida metapsilosis* and 4 of *Candida orthopsilosis*) and 16 of *Candida krusei*. All the isolates were characterized by sequencing of the internal transcribed spacer (ITS) region.

### 2. CLSI BMD method

CLSI BMD susceptibility testing was performed in accordance with the CLSI M27-A3 guidelines [12]. The following drugs and concentrations were tested: 5-flucytosine (0.125–64 µg/mL); amphotericin B, voriconazole, itraconazole (all 0.03–16 µg/mL); and fluconazole (0.125–64 µg/mL). Testing was done at 35°C (air) for 24 h. The MICs of all drugs were read following CLSI M27-A3 guideline [12].

### 3. ATB FUNGUS 3

An ATB FUNGUS 3 strip consists of 16 pairs of cupules including two growth control wells and five antifungal drugs at different concentrations: 5-flucytosine (4, 16 µg/mL), amphotericin B (0.5–16 µg/mL), fluconazole (1–128 µg/mL), itraconazole (0.125–4 µg/mL) and voriconazole (0.06–8 µg/mL). Testing on the ATB FUNGUS 3 strip was performed simultaneously with CLSI BMD. Following the manufacturer's instructions, a suspension with a turbidity of 2 McFarland was prepared and 20 µL of this suspension was transferred to an ampule of ATB FUNGUS 3 Medium. After this, 135 µL of the inoculated medium was transferred into each cupule.

**Figure 1:**
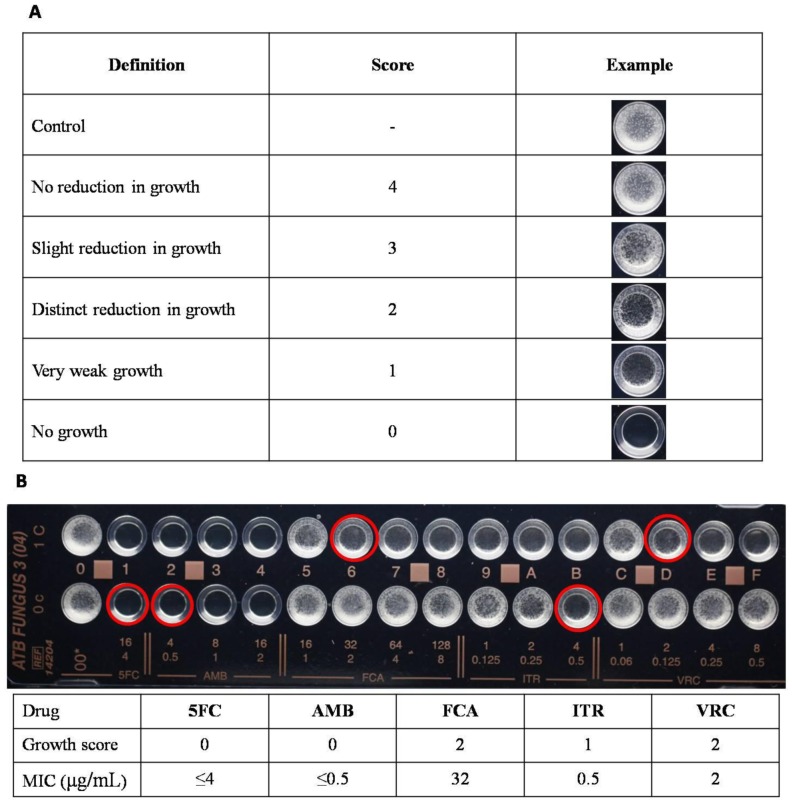
The interpretation of how to read the ATB FUNGUS 3 strips visually. **A.** Definitions of growth score. For amphotericin B, the MIC corresponds to the lowest concentration enabling complete growth inhibition (score = “0”). For 5-flucytosine, fluconazole, itraconazole and voriconazole, the MIC corresponds to the lowest concentration of the antifungal agent with which a score of “2”, “1”, “0” is obtained. **B.** An example showing how to read the MICs visually. Abbreviation: 5FC, 5-flucytosine; AMB, amphotericin B; FCA, fluconazole; ITR, itraconazole; VRC, voriconazole. Growth score: the score of the cupule marked with red circle.

After incubation at 35°C for 24 h, the strips were read both visually and automatically on the ATB Expression Bacteriology Analyzer automatic system (bioMérieux, La Balme-les Grottes, France). According to the manufacturer's instructions, the MICs were determined by the growth score for each of the cupules compared with the control cupules (Figure 1). For amphotericin B, the MIC corresponds to the lowest concentration enabling complete growth inhibition (score = “0”). For 5-flucytosine, fluconazole, itraconazole and voriconazole, the MIC corresponds to the lowest concentration of the antifungal agent with which a score of “2”, “1”, “0” is obtained.

### 4. Quality control

Quality control was ensured by testing the CLSI-recommended quality control strains *C. parapsilosis* ATCC 22019 and *C. krusei* ATCC 6528 for both CLSI BMD and ATB FUNGUS 3 [12].

### 5. Data analysis

The results obtained with the ATB FUNGUS 3 (both visual and automated readings) were compared with those of CLSI BMD after 24 h incubation. The results were considered to be in essential agreement (EA) when the ATB FUNGUS 3 results were within ±2 dilutions of the BMD reference value (eg. visual reading = 1 µg/mL, and automated reading = 4 µg/mL, defined as in EA; whilst visual reading = 1 µg/mL, automated reading = 8 µg/mL, defined as not in EA). The rates of EA (%) were the percentage of the isolates in EA with BMD. The results were considered to be in categorical agreement (CA) when the ATB FUNGUS 3 and CLSI BMD results fell within the same interpretive category (i.e., susceptible, susceptible dose dependent [SDD], intermediate, or resistant, depending on the drugs tested). If established CLSI M27-S4 interpretive breakpoints were available, they were used to determine CA. Epidemiological cutoff values (ECVs) were used for species/drugs without CLSI breakpoints (the susceptibility results are defined as either wide type [WT] or non-WT by ECVs) [13], [14]. Very major errors were identified when BMD indicated a resistant/non-WT result and the ATB FUNGUS 3 indicated a susceptible/WT one. Major errors were identified when BMD showed a susceptible/WT result while the ATB FUNGUS 3 showed the opposite. Minor errors were identified when one method recorded a susceptible or resistant result whilst the other recorded a SDD result.

### 6. Proposing a *Candida* species susceptibility testing algorithm

**Figure 2:**
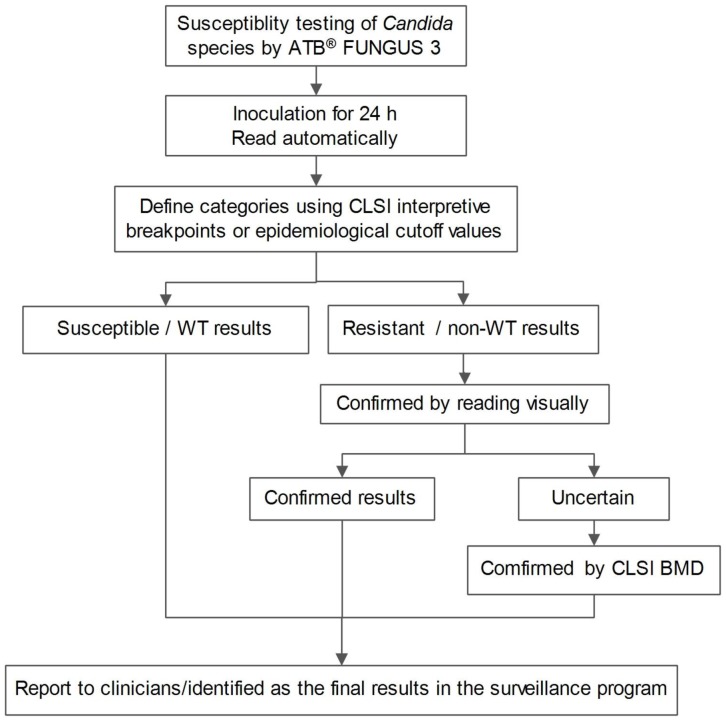
A proposed algorithm for susceptibility testing of *Candida* spp. using ATB FUNGUS 3. All the isolates are read automatically, but the *C. tropicalis* could be read visually according to the local epidemiological data. The categories were defined using the interpretive breakpoints by CLSI M27-S4 or epidemiological cut-off values (ECVs), and the isolates with any drug of resistant/non-WT results should be checked by reading visually. If there is still difficulty in reading or the resistance type is atypical, the susceptibility should be confirmed by CLSI BMD.

We developed an algorithm based on combination of ATB FUNGUS 3 automated and visual readings, which could be used when the isolates are identified to species level (Figure 2). Using this algorithm, the results are initially read by ATB automated reading system, and the isolates were put into categories [13], [14]. The isolates with non-resistant/WT results for all drugs can be reported as the final results, but those which show resistant results for any drug should be checked by visual reading. If there is still difficulty in the visual reading or the resistance type is atypical, then the susceptibility result should be confirmed by BMD assay.

### 7. Review of previously published antifungal susceptibility data

We reviewed a series of previous reports on ATB FUNGUS 3 by searching in China “Wanfang Med Online” (http://eng.med.wanfangdata.com.cn/) and PubMed (http://www.ncbi.nlm.nih.gov/pubmed) up to August 2014. The susceptibilities of *C. albicans* and *C. tropicalis* to fluconazole using ATB FUNGUS 3 (ATB Expression Bacteriology Analyzer automated read) in single centers, as well as ATB FUNGUS 3 (visual read) and the antifungal susceptibility data from surveillance programs were summarized in Table 1 for references [4], [6]–[11], [15].

**Table 1:** Reported susceptibilities of *C. albicans* and *C. tropicalis* to fluconazole using ATB visual and automated readings in single centers of China and susceptibilities by disc diffusion in global and national antifungal surveillance programs.

Method	Study site	Susceptibility to fluconazole (% resistant)		Reference
		*C. albicans*	*C. tropicalis*	
**Single center**				
ATB automated	a 3B-grade hospital in Shandong, China	60/270 (22.2)	15/60 (25.0)	Yu *et al.* 2007 (6)
ATB automated	a 2A-grade hospital in Xinjiang, China	19/143 (13.3)	10/19 (52.6)	Mi *et al.* 2008 (7)
ATB automated	a 3A-grade hospital in Liaoning, China	50/118 (42.3)	7/25 (26.9)	Wang *et al.* 2012 (8)
ATB visual	a 3A-grade hospital in Hainan, China	44/825 (5.3)	20/206 (9.7)	Huang *et al.* 2012 (9)
ATB visual	a 3A-grade hospital in Beijing, China	9/136 (6.6)	6/59 (10.2)	Li *et al.* 2013 (10)
ATB visual	an institution in Taiwan	13/388 (3.3)	10/171 (5.8)	Chen *et al.* 2009 (15)
**Surveillance program**				
Disc diffusion	133 institutions in the world (ARTEMIS)^a^	1800/128625 (1.4)	637/15546 (4.1)	Pfaller *et al.* 2010 (4)
Disc diffusion	24 institutions in Asia-Pacific region (ARTEMIS)^a^	259/28781 (0.9)	336/5178 (6.5)	Pfaller *et al.* 2010 (4)
Disc diffusion	12 institutions in China (CHIF-NET)^b^	1/282 (0.4)	7/123 (5.7)	Wang *et al.* 2012 (11)

a Five institutions from China were included.

b All 12 institutions were tertiary hospitals.

## Results

### 1. Data reviewed

As shown in Table 1, using the ATB automated reading system, the reported level of resistance to fluconazole reached as high as 42.3% for *C. albicans* and 52.6% for *C. tropicalis*, which is in sharp contrast to the results from visual readings (highest: 6.6% for *C. albicans* and 10.2% for *C. tropicalis*). Furthermore, published surveillance data for 12 institutions in China (CHIF-NET) showed fluconazole resistance rates of 0.4% (*C. albicans*) and 5.7% (*C. tropicalis*). The ARTEMIS global antifungal surveillance program also showed fluconazole resistance rates of only 1.4% for *C. albicans* and 4.1% for *C. tropicalis* (Table 1).

### 2. Essential agreement (EA) between ATB FUNGUS 3 and CLSI BMD

The overall EA between ATB visual readings and BMD was 99.1%. The highest concordance between the two methods for all the *Candida* species tested was observed for amphotericin B (100%) and 5-flucytosine (100%) (Table 2). Of all the *Candida* species tested, *C. glabrata* showed the lowest concordance between the two methods (Table 2 and 3). The overall EA between ATB automated readings and BMD was only 86.2%, which was significantly lower (P<0.05) than those of visual readings (99.1%). The highest agreement was observed for amphotericin B (99.5%) and 5-flucytosine (99.5%), but much lower EA for fluconazole (80.7%), voriconazole (77.5%), and itraconazole (73.4%). Most isolates with discrepant results had MICs of more than 2 dilutions higher than that of BMD for azole drugs. *C. tropicalis* isolates showed the lowest EA, with only 31.7% for fluconazole, 24.4% for voriconazole and 22.0% for itraconazole. In contrast, *C. parapsilosis* and *C. krusei* showed very high EA levels with BMD (Tables 2 and 3).

**Table 2:** Essential agreement (EA) and categorical agreement (CA) between ATB FUNGUS 3 (visual and automated readings) and CLSI BMD.

Species (no. of isolates tested)	Antifungal agent	Test method	EA no. (%)	CA no. (%)	Errors		
					Very major error	Major error	Minor error^b^
*C. albicans* (81)	fluconazole^a^	ATB visual	81 (100)	79 (97.5)	0 (0)	0 (0)	2 (2.5)
		ATB automated	71 (87.7)	73 (90.1)	0 (0)	8 (9.9)	0 (0)
	voriconazole^a^	ATB visual	81 (100)	81 (100)	0 (0)	0 (0)	0 (0)
		ATB automated	67 (82.7)	68 (84.0)	0 (0)	12 (14.8)	1 (1.2)
	itraconazole	ATB visual	81 (100)	80 (98.8)	1 (1.2)	0 (0)	-
		ATB automated	59 (72.8)	62 (76.5)	0 (0)	19 (23.5)	-
	amphotericin B	ATB visual	81 (100)	81 (100)	0 (0)	0 (0)	-
		ATB automated	81 (100)	81 (100)	0 (0)	0 (0)	-
	5-flucytosine	ATB visual	81 (100)	81 (100)	0 (0)	0 (0)	-
		ATB automated	81 (100)	81 (100)	0 (0)	0 (0)	-
*C. glabrata* (42)	fluconazole^a^	ATB visual	38 (90.5)	37 (88.1)	-	-	5 (11.9)
		ATB automated	38 (90.5)	36 (85.7)	-	-	6 (14.3)
	voriconazole	ATB visual	40 (95.2)	39 (92.9)	0 (0)	3 (7.1)	-
		ATB automated	38 (90.5)	30 (71.4)	0 (0)	12 (28.6)	-
	itraconazole	ATB visual	38 (90.5)	37 (88.1)	2 (4.8)	3 (7.1)	-
		ATB automated	38 (90.5)	27 (64.3)	1 (2.4)	14 (33.3)	-
	amphotericin B	ATB visual	42 (100)	42 (100)	0 (0)	0 (0)	-
		ATB automated	41 (97.6)	41 (97.6)	0 (0)	1 (2.4)	-
	5-flucytosine	ATB visual	42 (100)	42 (100)	0 (0)	0 (0)	-
		ATB automated	42 (100)	42 (100)	0 (0)	0 (0)	-
*C. tropicalis* (41)	fluconazole^a^	ATB visual	41 (100)	40 (97.6)	0 (0)	0 (0)	1 (2.4)
		ATB automated	13 (31.7)	17 (41.5)	0 (0)	23 (56.1)	1 (2.4)
	voriconazole^a^	ATB visual	41 (100)	38 (92.7)	0 (0)	0 (0)	3 (7.3)
		ATB automated	10 (24.4)	14 (34.2)	0 (0)	24 (58.5)	3 (7.3)
	itraconazole	ATB visual	41 (100)	41 (100)	0 (0)	0 (0)	-
		ATB automated	9 (22.0)	9 (22.0)	0 (0)	32 (78.0)	-
	amphotericin B	ATB visual	41 (100)	41 (100)	0 (0)	0 (0)	-
		ATB automated	41 (100)	41 (100)	0 (0)	0 (0)	-
	5-flucytosine	ATB visual	41 (100)	41 (100)	0 (0)	0 (0)	-
		ATB automated	41 (100)	41 (100)	0 (0)	0 (0)	-
*C. parapsilosis* (38)	fluconazole^a^	ATB visual	38 (100)	37 (94.7)	0 (0)	0 (0)	1 (5.3)
		ATB automated	38 (100)	30 (79.0)	0 (0)	1 (2.6)	7 (18.4)
	voriconazole^a^	ATB visual	38 (100)	38 (100)	0 (0)	0 (0)	0 (0)
		ATB automated	38 (100)	36 (94.7)	0 (0)	0 (0)	2 (5.3)
	itraconazole	ATB visual	38 (100)	38 (100)	0 (0)	0 (0)	-
		ATB automated	38 (100)	37 (97.4)	0 (0)	1 (2.6)	-
	amphotericin B	ATB visual	38 (100)	38 (100)	0 (0)	0 (0)	-
		ATB automated	38 (100)	38 (100)	0 (0)	0 (0)	-
	5-flucytosine	ATB visual	38 (100)	38 (100)	0 (0)	0 (0)	-
		ATB automated	38 (100)	38 (100)	0 (0)	0 (0)	-
*C. krusei* (16)	fluconazole	ATB visual	16 (100)	15 (93.7)	1 (6.3)	0 (0)	-
		ATB automated	16 (100)	15 (93.7)	1 (6.3)	0 (0)	-
	voriconazole^a^	ATB visual	16 (100)	11 (68.7)	0 (0)	0 (0)	5 (31.3)
		ATB automated	16 (100)	11 (68.7)	0 (0)	0 (0)	5 (31.3)
	itraconazole	ATB visual	16 (100)	16 (100)	0 (0)	0 (0)	-
		ATB automated	16 (100)	15 (93.7)	0 (0)	1 (6.3)	-
	amphotericin B	ATB visual	16 (100)	16 (100)	0 (0)	0 (0)	-
		ATB automated	16 (100)	16 (100)	0 (0)	0 (0)	-
	5-flucytosine	ATB visual	16 (100)	16 (100)	0 (0)	0 (0)	-
		ATB automated	15 (93.8)	16 (100)	0 (0)	0 (0)	-

a For these species/drugs, the CAs (%) were calculated based on breakpoints described in CLSI M27-S4; others were calculated based on ECVs.

b For species/drugs analyzed by ECVs, minor error was unavailable because there was no “SDD”.

**Table 3:** The number of strains for ATB visual and automated readings which were not in essential agreement (EA) with CLSI BMD.

Species	ATB visual readings^a^				ATB automated readings^a^				P value
	One drug	Two drugs	Three drugs	Agreement (%)^b^	One drug	Two drugs	Three drugs	Agreement^b^	visual vs. automated
*C. albicans* (81)	0	0	0	81 (100)	7	6	9	59 (72.8)	P<0.5
*C. glabrata* (42)	1	3	1	37 (88)	9	2	0	31 (85.6)	P>0.5
*C. tropicalis* (41)	0	0	0	41 (100)	3	2	28	8 (19.5)	P<0.5
*C. parapsilosis* (38)	0	0	0	38 (100)	0	0	0	38 (100)	P>0.5
*C. krusei* (16)	0	0	0	16 (100)	1	0	0	15 (93.8)	P<0.5

a The strains with susceptibility errors were divided into errors in one drug, two drugs and three drugs.

b The number of strains of which all the five drugs were in EA with CLSI BMD.

### 3. Categorical agreement (CA) between ATB FUNGUS 3 and CLSI BMD

The overall CA for the comparisons of the ATB visual reading results with the BMD results was 97.5%, and 95.4% and 95.0% for fluconazole and voriconazole, respectively (Table 2). In contrast, the overall CA between the ATB automated readings and the BMD results was only 84.0%, and 78.4% and 72.9% for fluconazole and voriconazole, respectively. The highest major error rate was observed for susceptibility of *C. tropicalis* to either fluconazole (23 of 41 isolates studied, 56.1%) or voriconazole (24 of 41, 58.5%), followed by *C. albicans* (8 of 81 [9.9%] isolates for fluconazole and 12 of 81 [14.8%] to voriconazole (Table 2).

### 4. Validation of the proposed algorithm

Based on this algorithm, all *C. tropicalis* isolates can be directly read visually because of poor accuracy by the ATB automated reading system. However, the local prevalence of *C. tropicalis* should be considered when choosing this proposal. When we applied the proposed algorithm to the 218 isolates in the present study as shown in Table 4, the algorithm offered a very good balance between accuracy and automation. As can be seen, the proposed testing algorithm is practical, labor and time saving for *Candida* susceptibility testing in large surveillance programs (Table 4).

**Table 4:** Comparison of four strategies for susceptibility testing of 218 isolates of *Candida* using ATB FUNGUS 3.

Strategy	No. isolates read automatically	No. isolates read visually^a^	Final agreement^b^ (%)
All read automatically	218	0	151/218 (69.3)
All read visually	0	218	216/218 (99.0)
Proposed algorithm	218	88 “resistant/non-WT”	213/218 (97.7)
Proposed algorithm (*C. tropicalis* read visually directly)	177	93 (41 *C. tropicalis*+52 “resistant/non-WT”)	213/218 (97.7)

a The number of re-checked strains in proposed algorithm is a little high because all the resistant isolates of the five *Candida* species in CHIF-NET 10 were included in the present study.

b The number of the strains of which the susceptibilities to the five drugs were all in EA with BMD.

## Discussion

ATB FUNGUS 3 is the most commonly used commercialized antifungal susceptibility test method in China, and is also widely used in parts of Africa, South America and Europe [16]–[19]. A former version (ATB FUNGUS 2) of this current system has been evaluated by Torres-Rodriguez *et al.* [17]. In agreement to our findings, lower agreement with BMD was observed for fluconazole and itraconazole, particularly with *C. tropicalis* and *C. albicans*. [17].

In the present evaluation, ATB visual readings showed good concordance with BMD, whilst a high pseudo resistant rate (defined as major errors) to azole drugs was observed for ATB automated readings. The potential explanation for the high major errors with the ATB automated readings is the trailing effect of azoles. We found that *C. tropicalis* and *C. albicans* showed obvious trailing growth in testing with azole drugs. The activation of calcineurin and altered regulation of genes mediating resistance could partially explain the trailing phenomenon [20], [21].

The major errors in azole drug susceptibility testing by ATB automated readings are a cause for concern for several reasons. Firstly, the very high pseudo-resistance may result in misleading clinical antifungal drug selection and increased costs for antifungal drugs. Secondly, national and international surveillance studies and epidemiological comparison studies may potentially get misleading results and conclusions if they simply used the ATB automated reading data - generally will over-estimate the China azoles antifungal resistance rates. Thirdly, the unwarranted use of alternative antifungal agents may promote further resistance in these agents, making future drug selection difficult [22]. Therefore, the ATB visual reading is generally recommended.

The present study highlights the flaws encountered in commercial automated identification and susceptibility testing systems especially in developing countries. Very recently, similar problems were reported by Chowdhary *et al.* in Asia, where VITEK 2 (bioMérieux, Marcy I'Etoile, France) misidentified 10 *Candida auris* isolates as *Candida haemulonii* and 2 as *Candida famata* [23], [24]. The problems highlighted the need for an improvement and validation of the automated systems as well as alerting laboratory staff of the potential errors. Before the improvement of the automated reading system, we developed an algorithm to make good use of the ATB reading system, which is especially practical for surveillance programs.

Generally, the ATB FUNGUS 3 (visual reading) is a simple and accurate method for the determination of MICs for *Candida* spp., which is suitable for clinical microbiology use in developing countries like in Asia, Africa and South America. The present study served as an important experience in ATB FUNGUS 3 practical use for other countries where it is routinely used.

## References

[1] Falagas ME, Apostolou KE, Pappas VD (2006) Attributable mortality of candidemia: a systematic review of matched cohort and case-control studies. Eur J Clin Microbiol Infect Dis 25:419–425. 16773391 10.1007/s10096-006-0159-2

[2] Miceli MH, Diaz JA, Lee SA (2011) Emerging opportunistic yeast infections. Lancet Infect Dis 11:142–151. 21272794 10.1016/S1473-3099(10)70218-8

[3] Pfaller MA, Diekema DJ (2007) Epidemiology of invasive candidiasis: a persistent public health problem. Clin Microbiol Rev 20:133–163. 17223626 10.1128/CMR.00029-06 PMC1797637

[4] Pfaller MA, Diekema DJ, Gibbs DL, Newell VA, Ellis D, et al (2010) Results from the ARTEMIS DISK Global Antifungal Surveillance Study, 1997 to 2007: a 10.5-year analysis of susceptibilities of *Candida* species to fluconazole and voriconazole as determined by CLSI standardized disk diffusion. J Clin Microbiol 48:1366–1377. 20164282 10.1128/JCM.02117-09 PMC2849609

[5] Pappas PG, Kauffman CA, Andes D, Benjamin DJ, Calandra TF, et al (2009) Clinical practice guidelines for the management of candidiasis: 2009 update by the Infectious Diseases Society of America. Clin Infect Dis 48:503–535. 19191635 10.1086/596757 PMC7294538

[6] Yu JH, Diao YL, Zhu SY (2007) [Distribution and drug susceptibility analysis of yeast-like fungal infection]. Clin J Med Offic 35:268–269 (in Chinese).

[7] Mi RS, Ni SRH, Tian G (2008) [Susceptibility analysis of 167 *Candida* spp. from sputum species]. Lab Med Clin 19:1173–1174 (in Chinese).

[8] Wang W, Shi L, Xue H, Wu LX, Zhao YH (2012) [Infection characters and drug-sensitivity of yeast-like fungi in clinical practice]. Chin Mod Med 19:98–99 (in Chinese).

[9] Huang H, Wu DR, Han XS, Zhou W, Zhang DX (2012) [Identification and antifungal susceptibility tests of 1312 strains of yeast]. Practical Prevent Med 19:1536–1537 (in Chinese).

[10] Li F, Wu L, Cao B, Zhang Y, Li X, et al (2013) Surveillance of the prevalence, antibiotic susceptibility, and genotypic characterization of invasive candidiasis in a teaching hospital in China between 2006 to 2011. BMC Infect Dis 13:353. 23899244 10.1186/1471-2334-13-353 PMC3733982

[11] Wang H, Xiao M, Chen SC, Kong F, Sun ZY, et al (2012) In vitro susceptibilities of yeast species to fluconazole and voriconazole as determined by the 2010 National China Hospital Invasive Fungal Surveillance Net (CHIF-NET) study. J Clin Microbiol 50:3952–3959. 23035204 10.1128/JCM.01130-12 PMC3502960

[12] Clinical and Laboratory Standards Institute (2008) Reference method for broth dilution antifungal susceptibility testing of yeasts; Approved standard, 3th ed, M27-A3. Clinical and Laboratory Standards Institute, Wayne, PA.

[13] Clinical and Laboratory Standards Institute (2012) Reference method for broth dilution antifungal susceptibility testing of yeasts; Fourth informational supplement, 4th ed, M27-S4. Clinical and Laboratory Standards Institute, Wayne, PA.

[14] Pfaller MA, Diekema DJ (2012) Progress in antifungal susceptibility testing of *Candida* spp. by use of Clinical and Laboratory Standards Institute broth microdilution methods, 2010 to 2012. J Clin Microbiol 50:2846–2856. 22740712 10.1128/JCM.00937-12 PMC3421803

[15] Chen P, Lo H, Wu C, Lee H, Chang C, et al (2011) Species distribution and antifungal susceptibility of blood *Candida* isolates at a tertiary hospital in southern Taiwan, 1999–2006. Mycoses 54:e17–e23. 20028463 10.1111/j.1439-0507.2009.01818.x

[16] Horta JA, Staats CC, Casali AK, Ribeiro AM, Schrank IS, et al (2002) Epidemiological aspects of clinical and environmental *Cryptococcus neoformans* isolates in the Brazilian state Rio Grande do Sul. Med Mycol 40:565–571. 12521120 10.1080/mmy.40.6.565.571

[17] Torres-Rodriguez JM, Alvarado-Ramirez E (2007) In vitro susceptibilities to yeasts using the ATB FUNGUS 2 method, compared with Sensititre Yeast One and standard CLSI (NCCLS) M27-A2 methods. J Antimicrob Chemother 60:658–661. 17623690 10.1093/jac/dkm247

[18] Eraso E, Ruesga M, Villar-Vidal M, Carrillo-Munoz AJ, Espinel-Ingroff A, et al (2008) Comparative evaluation of ATB Fungus 2 and Sensititre YeastOne panels for testing in vitro *Candida* antifungal susceptibility. Rev Iberoam Micol 25:3–6. 18338919 10.1016/s1130-1406(08)70002-0

[19] Ben AJ, Saghrouni F, Nouri S, Geith S, Khammari I, et al (2012) Neonatal invasive candidiasis in Tunisian hospital: incidence, risk factors, distribution of species and antifungal susceptibility. Mycoses 55:493–500. 22448706 10.1111/j.1439-0507.2012.02189.x

[20] Sanglard D, Ischer F, Marchetti O, Entenza J, Bille J (2003) Calcineurin A of *Candida albicans*: involvement in antifungal tolerance, cell morphogenesis and virulence. Mol Microbiol 48:959–976. 12753189 10.1046/j.1365-2958.2003.03495.x

[21] Lee MK (2004) Drug resistance genes and trailing growth in *Candida albicans* isolates. J Antimicrob Chemother 53:217–224. 14688046 10.1093/jac/dkh040

[22] Hou D, Wang Q, Jiang C, Tian C, Li H, et al (2014) Evaluation of the short-term effects of antimicrobial stewardship in the intensive care unit at a tertiary hospital in China. PLoS ONE 9:e101447. 25000225 10.1371/journal.pone.0101447 PMC4084822

[23] Chowdhary A, Sharma C, Duggal S, Agarwal K, Prakash A, et al (2013) New clonal strain of *Candida auris*, Delhi, India. Emerg Infect Dis 19:1670–1673. 24048006 10.3201/eid1910.130393 PMC3810747

[24] Chowdhary A, Anil Kumar V, Sharma C, Prakash A, Agarwal K, et al (2014) Multidrug-resistant endemic clonal strain of *Candida auris* in India. Eur J Clin Microbiol Infect Dis 33:919–926. 24357342 10.1007/s10096-013-2027-1

